# Contrast-enhanced ultrasound of benign and malignant liver lesions in children

**DOI:** 10.1007/s00247-021-04976-2

**Published:** 2021-05-12

**Authors:** Cheng Fang, Sudha A. Anupindi, Susan J. Back, Doris Franke, Thomas G. Green, Zoltan Harkanyi, Jörg Jüngert, Jeannie K. Kwon, Harriet J. Paltiel, Judy H. Squires, Vassil N. Zefov, M. Beth McCarville

**Affiliations:** 1 Department of Radiology, King’s College Hospital, Denmark Hill, London, SE5 9RS UK; 2 Department of Radiology, Perelman School of Medicine, Children’s Hospital of Philadelphia, University of Pennsylvania, Philadelphia, PA USA; 3 Department of Pediatric Kidney, Liver and Metabolic Diseases, Hannover Medical School, Hannover, Germany; 4 Department of Radiology, Crouse Hospital, Syracuse, NY USA; 5 Department of Radiology, Heim Pál National Pediatric Institute, Budapest, Hungary; 6 Department of Pediatrics, Friedrich-Alexander University Erlangen–Nürnberg, Erlangen, Germany; 7 Department of Radiology, University of Texas Southwestern Medical Center, Dallas, TX USA; 8 Department of Radiology, Harvard Medical School, Boston Children’s Hospital, Boston, MA USA; 9 Department of Radiology, Children’s Hospital of Pittsburgh, University of Pittsburgh Medical Center, Pittsburgh, PA USA; 10 Department of Radiology, Dubai Health Authority, Latifa Women and Children Hospital, Dubai, UAE; 11 Department of Diagnostic Imaging, St. Jude Children’s Research Hospital, Memphis, TN USA

**Keywords:** Benign, Children, Contrast-enhanced ultrasound, Lesion, Liver, Malignant, Ultrasound, Ultrasound contrast agents

## Abstract

Contrast-enhanced ultrasound (CEUS) is increasingly being used in children. One of the most common referrals for CEUS performance is characterization of indeterminate focal liver lesions and follow-up of known liver lesions. In this setting, CEUS is performed with intravenous administration of ultrasound contrast agents (UCAs). When injected into a vein, UCA microbubbles remain confined within the vascular network until they dissipate. Therefore, visualization of UCA within the tissues and lesions corresponds to true blood flow. CEUS enables continuous, real-time observation of the enhancement pattern of a focal liver lesion, allowing in most cases for a definite diagnosis and obviating the need for further cross-sectional imaging or other interventional procedures. The recent approval of Lumason (Bracco Diagnostics, Monroe Township, NJ) for pediatric liver CEUS applications has spurred the widespread use of CEUS. In this review article we describe the role of CEUS in pediatric liver applications, focusing on the examination technique and interpretation of main imaging findings of the most commonly encountered benign and malignant focal liver lesions. We also compare the diagnostic performance of CEUS with other imaging modalities for accurate characterization of focal liver lesions.

**Supplementary Information:**

The online version contains supplementary material available at 10.1007/s00247-021-04976-2.

## Introduction

Liver applications of contrast-enhanced ultrasound (CEUS) are increasingly reported in children. Focal liver lesions can be encountered either incidentally in asymptomatic children during routine abdominal imaging examinations (usually by US), in staging or follow-up scans of children with cancer, or in the setting of surveillance programs for chronic liver disease or other conditions that predispose to malignancy (e.g.*,* overgrowth syndromes). Common benign solid liver lesions in the pediatric population include infantile and congenital hemangiomas and focal nodular hyperplasia, whereas hepatic adenoma and regenerative nodular hyperplasia occur infrequently [1]. Occasionally, pseudo-lesions such as focal fat sparing or deposition mimic liver lesions and warrant further investigation. The most common primary malignant tumor among children younger than 5 years is hepatoblastoma, and among those 15–19 years old is hepatocellular carcinoma [2, 3].

Although the first-line imaging modality used for the detecting focal liver lesions is usually gray-scale US coupled with color/power Doppler techniques, its accuracy in characterizing the nature of a lesion is limited because of the nonspecific echo pattern of several lesions (predominantly small or deeply located lesions). Accuracy of Doppler US is also affected by artifacts (e.g.*,* respiratory or cardiac motion artifacts, or shadowing from bowel gas or ribs) and technical limitations (e.g.*,* inappropriate color scale settings). In most cases, US is followed by contrast-enhanced cross-sectional imaging, CT or MRI, the latter being preferable in pediatric patients for complete assessment of focal liver lesions.

Contrast-enhanced ultrasound (CEUS) is increasingly integrated into imaging algorithms for detecting and characterizing focal liver lesions. CEUS is particularly well-suited for the pediatric population because it is free of ionizing radiation, does not require sedation, lacks toxicity (renal, hepatic and cardiac) and can be performed in the presence of caregivers or even at the bedside. Performing CEUS simultaneously with the initial unenhanced US allows in most cases a definitive diagnosis and complete assessment of a lesion. This is particularly important because it alleviates the anxiety of children and families and facilitates the workflow in busy radiology departments.

Three currently available ultrasound contrast agents (UCAs) have been used for liver imaging in children: SonoVue (Bracco Imaging SpA, Milan, Italy), which is marketed as Lumason (Bracco Diagnostics, Monroe Township, NJ) in the United States; Optison (GE Healthcare, Princeton, NJ); and Definity (Lantheus, North Billerica, MA). In April 2016, the United States Food and Drug Administration (FDA) approved Lumason for intravenous liver applications in children. Currently, all other UCAs that are used are off-label for liver investigations in children.

Numerous CEUS studies conducted in adults and a growing number of CEUS studies in children have demonstrated a high safety profile for the intravenous use of UCAs [4–21]. In 2006, the largest prospective multicenter CEUS study, comprising 231,888 adults, reported an incidence of 0.13% for all adverse reactions and 0.01% for serious adverse reactions with UCA administration [22]. This is lower than what has been reported with iodine-based contrast media, for which overall acute allergic-like reaction frequency ranges from 0.18% to 0.46% in people who receive low-osmolality contrast media [23–27]. The allergic-like adverse reactions for gadolinium range 0.004% to 0.70%, with the added risks of nephrogenic systemic fibrosis and tissue deposition [28–32].

The reported indications of CEUS for liver imaging in children include detection and characterization of indeterminate focal liver lesions. These lesions are either discovered incidentally or during surveillance imaging examinations, usually by US. However, CEUS can also be used as an adjunct or problem-solving tool in cases when MRI or CT are inconclusive or technically limited (e.g., when there are motion artifacts). In addition, CEUS has been used in the follow-up of known liver lesions that are either treated conservatively or have undergone interventional procedures (e.g., post chemotherapy or ablation) [33–35]. Other CEUS applications for liver imaging include trauma, post-liver transplantation, and interventional procedures. These are covered in detail in other articles in this supplement [36–38].

In this article, we discuss the role of CEUS in the characterization of focal liver lesions in children. We review the CEUS examination technique and the typical imaging features of the most common benign and malignant pediatric liver lesions. We also discuss the diagnostic performance of CEUS compared to that of other imaging modalities.

## Contrast-enhanced ultrasound examination technique

### Patient preparation

No sedation, general anesthesia or prior blood tests are required. Where available and when needed, a child life specialist might facilitate the study by helping the child to remain calm and cooperate with the examination.

### Ultrasound contrast agent dose

The FDA-approved dose of Lumason for pediatric liver applications is 0.03 mL/kg, up to a maximum of 2.4 mL per single bolus dose. This dose can be given twice during an examination, if needed. Variable doses of SonoVue for liver imaging in the pediatric population have been published. These doses were either adjusted based on the child’s age and body weight or were arbitrarily selected. Centers have employed a range of approaches to dose calculation. One center uses an age-based approach to doses of SonoVue, so that children younger than 6 years receive 0.6 mL, 6–12 years 1.2 mL and older than 12 years 2.4 mL [7, 8]. Another center uses a different age-based approach so that children receive 0.1 mL of SonoVue per each year of age [17]. A third center uses a weight-based dose so that children weighing up to 24 kg receive 0.1 mL/kg, and those weighing more than 24 kg receive 2.4 mL [13]. Other centers use doses ranging from 0.1 mL to 4.8 mL [6, 9–12] (Table 1; [6–9, 12, 13, 15, 21, 39]).

**Table 1:** Original research, pediatric exclusive contrast-enhanced ultrasound (CEUS) studies of focal liver lesions using SonoVue/Lumason

Reference	Ultrasound contrast agent dose	CEUS exams for focal liver lesions (*n*)^a^
El-Ali 2020 [21]	0.03 mL/kg	10
Fang 2019 [7]	<1 y: 0.6 mL; ≥1 y: 1.2–2.4 mL	30
Yusuf 2017 [8]	<6 y: 0.6 mL; 6–12 y: 1.2 mL; ≥12 y: 2.4 mL	147
Torres 2017 [13]	0.1 mL/kg (maximum 2.4 mL)	Native liver: 92 Transplant liver:17
Knieling 2016 [15]	<20 kg: 0.4±0.3 mL; >20 kg: 1.0±0.4 mL	41^c^
Pschierer 2015 [12]	0.5–3 mL	49
Piskunowicz 2015 [6]^b^	0.1–1.8 mL	48^d^
Jacob 2013 [9]	1.2–2.4 mL	44
Stenzel 2013 [39]	0.1 mL/year of age	14^e^

*y* years

^a^While some studies included information about CEUS performed to assess non-focal lesions (e.g., portal vein or biliary assessment), only the information about focal liver lesions is summarized in this table

^b^This study was prospective; all other studies were retrospective

^c^Study included a total of 55 CEUS examinations; 41/55 were performed for focal liver lesions

^d^The study included a total of 161 CEUS examinations; 48/161 were performed for focal liver lesions

^e^The study included a total of 39 CEUS examinations; 14/39 were performed for focal liver lesions

Optison has been used for pediatric abdominal applications of intravenous (IV) CEUS under an FDA-approved investigational new drug application. In the preliminary safety and feasibility part of the study, the selected dose of Optison was initially based on body surface area, beginning at a very low dose of 0.125 mL/m^2^ and escalating in 0.075 mL/m^2^ increments to a maximum of 0.350 mL/m^2^ [35]. Later, dosing of this agent was changed to a weight-based approach, such that children <20 kg received 0.3 mL and those ≥20 kg received 0.5 mL of Optison [34]. Definity has been used in a few children for IV CEUS in abdominal applications, but no specific dosing data have been reported [34].

It is generally accepted that the dose can be adjusted according to the sensitivity of the US machine contrast software and transducer frequency because higher-frequency transducers require more UCA [40].

### Pre-contrast scan

First, a baseline US scan is performed. In this scan the operator should determine the position of the child and the sonographic window that will allow an optimal view of the focal liver lesion at all times.

### Post-contrast scan

The CEUS examination should be performed using contrast-specific low mechanical index imaging (i.e. <0.3) [41]. For the operator to constantly monitor the lesion throughout the examination, a dual screen with simultaneous display of the reference gray-scale US image alongside the contrast-enhanced image is preferred, with or without simultaneous caliper display on both screens.

Contrast-enhanced US liver imaging should be performed with a curved-array transducer at a frequency of 3–10 MHz for infants and small children, whereas lower-frequency transducers of 1–5 MHz can be used in older or obese children to maximize contrast signals. Higher-frequency linear transducers (e.g., 2–9 MHz) can be used for small superficial liver lesions.

The child should be in a comfortable position, and scanned during free, shallow breathing. If the child is able, breath holds can be helpful to visualize lesions near the liver dome or beneath the ribs. The examination requires two people, one to scan and one to administer the contrast agent. When optimal visualization of the target lesion is established, the UCA is given and then the IV tubing is flushed with 3–10 mL of normal saline (0.9%), pushing the residual contrast agent into circulation.

For the IV, the cannula is usually 24-gauge or larger and is placed in the antecubital fossa. However, smaller or more peripherally inserted cannulas can also be used, particularly in infants and small children, if this IV access is the only one available. A central line can be used if a peripheral one is not available.

## Interpretation of imaging findings

When interpreting the enhancement features of a focal liver lesion, the examiner should have in mind the basic principles described in the following sections.

### Ultrasound contrast agent pharmacodynamics

Ultrasound contrast agents comprise microspheres that approximate the size of red blood cells, so they remain strictly confined within the vessels. In contradistinction, iodine-based contrast agents and gadolinium comprise particles much smaller than red blood cells and these particles can freely diffuse across the vascular membrane into the interstitial space. These inherent differences between the pharmacodynamic properties of UCAs and CT/MRI contrast agents result in certain disparities between the enhancement patterns of focal liver lesions seen on CEUS and those seen on contrast-enhanced CT and MRI. On CEUS, any enhancement observed represents true intravascular blood flow within vascular components of a lesion. On CT/MRI, both intravascular and extracellular pools of contrast agent contribute to parenchymal and lesional enhancement.

### Contrast-enhanced ultrasound enhancement phases

The liver has a dual blood supply from the hepatic artery and main portal vein, contributing to approximately 25% and 75% of blood flow to the liver, respectively. As a result of this dual vascular supply, after intravenous contrast administration, different vascular structures in the normal liver reach peak enhancement at different times. Specifically, the hepatic arteries reach peak enhancement first, followed by the portal veins, and then the hepatic veins. This allows the distinction of three enhancement phases: the arterial phase, the portal venous phase and the late phase [42]. These three phases represent a continuum, with one phase gradually changing to the other. However, for clinical utility and communication purposes the following time intervals have been defined: the arterial phase starts 10–20 s after UCA injection and lasts up to 35–40 s; the portal venous phase starts at 30–45 s after UCA injection and lasts up to 120 s; the late phase starts after 120 s until there is complete clearance of UCA from the circulation at approximately 4–6 min [42–44].

Contrast-enhanced ultrasound allows for real-time observation and recording of this continuum of enhancement through all phases from the moment of contrast injection until contrast is eliminated from the circulation — without the risk of ionizing radiation. In addition, UCA injection can be repeated if there is a need to confirm the findings, to clarify equivocal or inconclusive findings from the first CEUS examination, or to evaluate an additional lesion in the arterial phase [45].

Unlike CEUS, contrast-enhanced CT entails intermittent static image acquisition in standardized time intervals. Depending on the purpose of the investigation (i.e. whether it is an initial study or a follow-up study), a multiphase contrast-enhanced CT scan is performed for characterizing focal liver lesions, particularly in adults. At least two imaging phases are required for the initial characterization of most liver lesions; the arterial and portal venous enhancement phases are typically acquired at 20 s and 40–60 s, respectively. For specific indications, an unenhanced study can be performed first (e.g., for intralesional hemorrhage, or detection of calcifications), and a late parenchymal phase at 90 s might also be acquired, although the former is generally not recommended in children [46].

Because of the significant burden of ionizing radiation involved in a multiphase CT scan, contrast-enhanced MRI is the imaging modality of choice for liver lesion characterization in many pediatric institutions [46]. With MRI, pre-contrast imaging is followed by dynamic post-contrast image acquisition in the arterial phase, the portal venous phase, and the delayed or equilibrium phases (2–5 min after injection). However, additional delayed images after 2–5 min can help characterize certain lesions such as hemangiomas and vascular malformations. If a hepatobiliary contrast agent is used, the hepatobiliary phase (typically at 20 min after injection with gadoxetate, and approximately 45 min with gadobenate) is also acquired [27, 47].

### Contrast-enhanced ultrasound enhancement terminology

The conspicuity of a focal liver lesion during CEUS depends on the differential enhancement between the lesion and background normal hepatic parenchyma. Comparison of the lesion to the normal hepatic parenchyma is made at each enhancement phase. A lesion is characterized as non-enhancing if there is no contrast uptake. If during an enhancement phase there is equal, reduced or greater enhancement compared to the adjacent liver at the same depth, the lesion is characterized as iso-, hypo- or hyperenhancing, respectively [43]. Washout refers to the visible reduction of UCA signal in portions of or in the entire lesion that follows its initial maximum enhancement. Washout of a lesion is compared to the adjacent liver parenchyma during any phase. The presence of contrast washout is the main imaging feature of malignancy, whereas its absence suggests benignity [48]. The purely intravascular nature of UCA allows for better and more consistent determination of the washout feature. However, a child’s comorbidities can affect interpretation of the washout of a focal liver lesion, e.g.*,* underlying cirrhosis might be associated with poor enhancement of the background liver parenchyma and therefore less discrete washout.

### Contrast-enhanced ultrasound scanning technique

Depending on the size and location of the lesion, transverse, sagittal or oblique images can be obtained. The examiner should select the scanning plane in which the target lesion is visualized in its entirety, if possible, and at the shortest possible distance from the transducer. In addition, in that same single imaging plane the lesion should be visualized along with a substantive part of the surrounding normal liver parenchyma, including adjacent vascular structures where possible.

The timer should be started when the UCA is injected and should be visible throughout the study. The operator should hold the transducer stationary over the area of interest, observing and recording in a video clip the perfusion of the UCA within the lesion from the moment of injection and for at least 60 s. This continuous phase is followed by intermittent imaging of the target lesion at 5–10 s, repeated every 30–60 s past 5 min or until the contrast agent has completely washed out from the rest of the liver [43, 49]. Additionally, after the first 60 s, the examiner can intermittently scan through the rest of the liver in standard transverse and sagittal planes to look for additional lesions.

Care must be taken to avoid prolonged insonation over the region of interest, which can lead to microbubble destruction by acoustic energy. The extent of microbubble destruction depends on how long the probe is stationary, and the transducer frequency; UCA microbubbles in the nearfield are destroyed most quickly. If microbubble destruction occurs, it is possible to make erroneous conclusions by misinterpreting this loss of contrast signal as contrast washout [50]. If a second UCA dose is required, the examiner should ensure that the first UCA dose has been cleared from the circulation by allowing 10 min to pass between the first and second injections, or until contrast agent is no longer visible. Alternatively, one can use the flash technique with high mechanical index to quickly burst the microbubbles and clear the imaging field.

## Interpretation of contrast-enhanced ultrasound findings in benign liver lesions

### Infantile hemangioma

Infantile hemangioma is a benign vascular tumor of the liver and is the most common hepatic tumor in infants (<6 months) with female predominance of 3:1 [51]. It is composed of large endothelial-lined vascular channels, similar to infantile hemangiomas elsewhere in the body [52]. The natural course is typical, with presentation shortly after birth, rapid proliferation in the first 6–12 months of age, and spontaneous regression and slow involution in the first 2–3 years of age [53]. These lesions are usually discovered during the proliferative phase because of associated hepatomegaly and abdominal distention, or during screening of infants with >5 cutaneous infantile hemangiomas. If there is extensive high-flow arteriovenous shunting within the lesion, high-output congestive heart failure can occur. Infantile hemangiomas are classified by subtypes as focal, multifocal and diffuse abnormalities [51, 54].

On gray-scale US, depending on the specific subtype, imaging appearances vary. Small, focal infantile hemangiomas appear as well-defined, predominantly hypoechoic lesions. Larger lesions tend to be heterogeneous, predominantly hypoechoic or of mixed echogenicity, with echogenic areas corresponding to the interfaces between the walls of the vascular channels and fibrosis. Anechoic areas correspond to the vascular channels or represent areas of necrosis. The diffuse subtype demonstrates near-complete replacement of the hepatic parenchyma with innumerable well-circumscribed nodules. Color Doppler US reveals enlarged peri- and intralesional arteries, veins and shunts [55, 56]. Calcifications are seen in up to 36% of these children, usually with tumor involution [56].

**Fig. 1:**
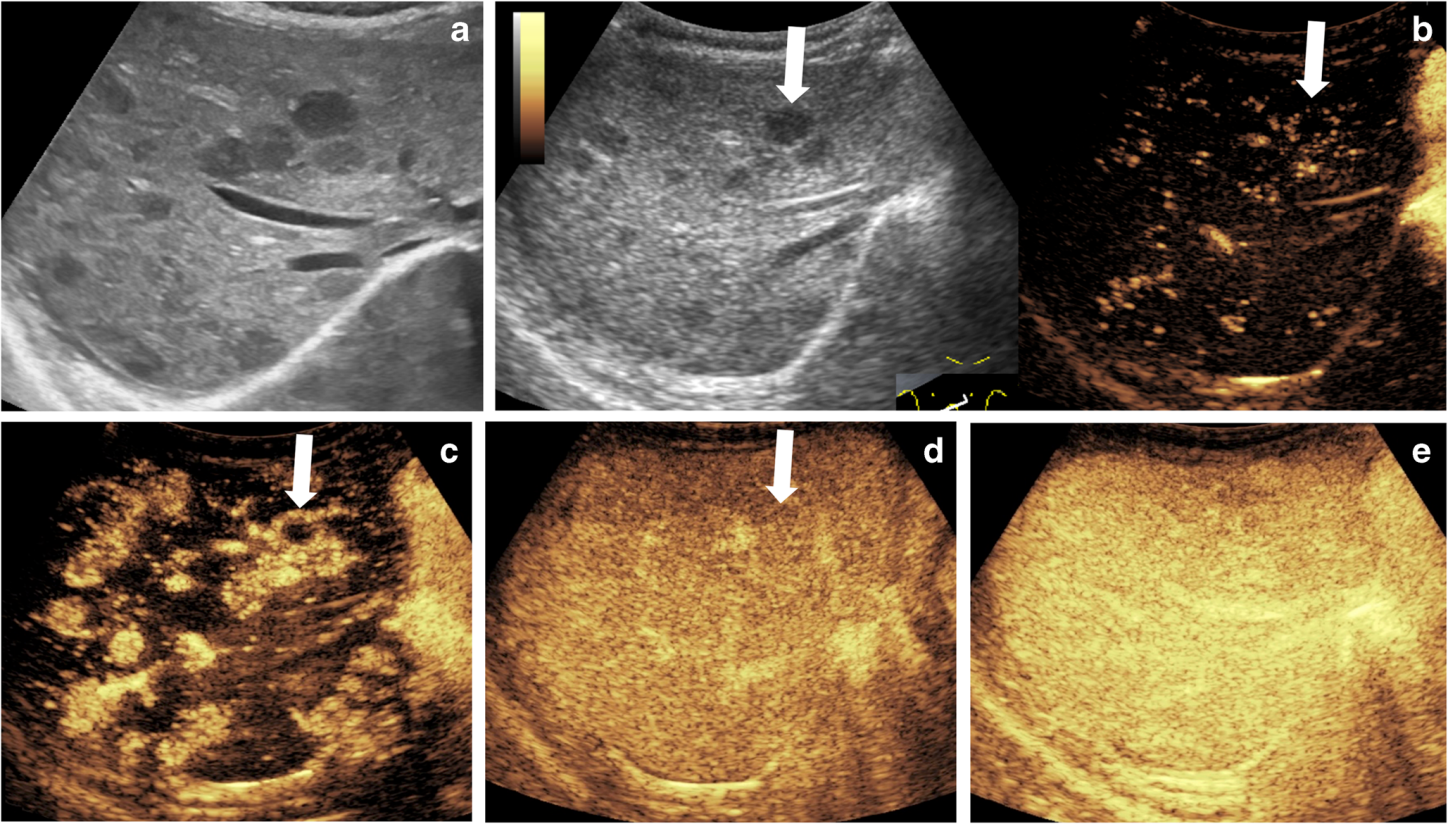
Incidental liver hemangiomatosis in a 4-month-old boy. **a** Transverse gray-scale US shows multiple hypoechoic lesions throughout the liver. **b–e** Transverse contrast-enhanced ultrasound (CEUS) of the liver. Dual display of the gray-scale (*left*) and contrast-enhanced (*right*) image at 8 s (**b**) and contrast-only CEUS images at 10 s (**c**), 22 s (**d**) and 1 min 33 s (**e**) after contrast administration. Early peripheral enhancement is exemplified in the largest lesion (*arrows*), followed by centripetal enhancement and nearly complete and homogeneous enhancement of all lesions similar to the background hepatic parenchyma. No washout is noted in any of the lesions (**e**)

The typical CEUS appearances of infantile hemangiomas are expected to mimic the well-described enhancement patterns reported for CT and MRI: in the early arterial phase, there is peripheral nodular (circular or semi-circular) enhancement; in the portal venous and late phases, there is centripetal progression and complete iso- or hyperenhancement of the lesion compared to the adjacent liver parenchyma, with subtle late washout possible (Fig. 1) [4, 5, 21, 57].

### Congenital hemangioma

Congenital hemangioma is also a benign vascular lesion, with distinctly different histological and clinical features from those of infantile hemangioma. It occurs with equal frequency in female and male infants. Unlike infantile hemangiomas, a congenital hemangioma is typically present at birth but does not grow after birth [52, 58, 59]. Depending on the involution rate, congenital hemangiomas are divided into three categories: rapidly involuting congenital hemangiomas that begin to involute soon after birth (most of these have significantly decreased in size by 2 years of age); non-involuting congenital hemangiomas that do not involute or decrease in size over time; and partially involuting congenital hemangioma, in which the involution occurs during the first 12 months of age, at which point the rate of involution decreases over time [59].

**Fig. 2:**
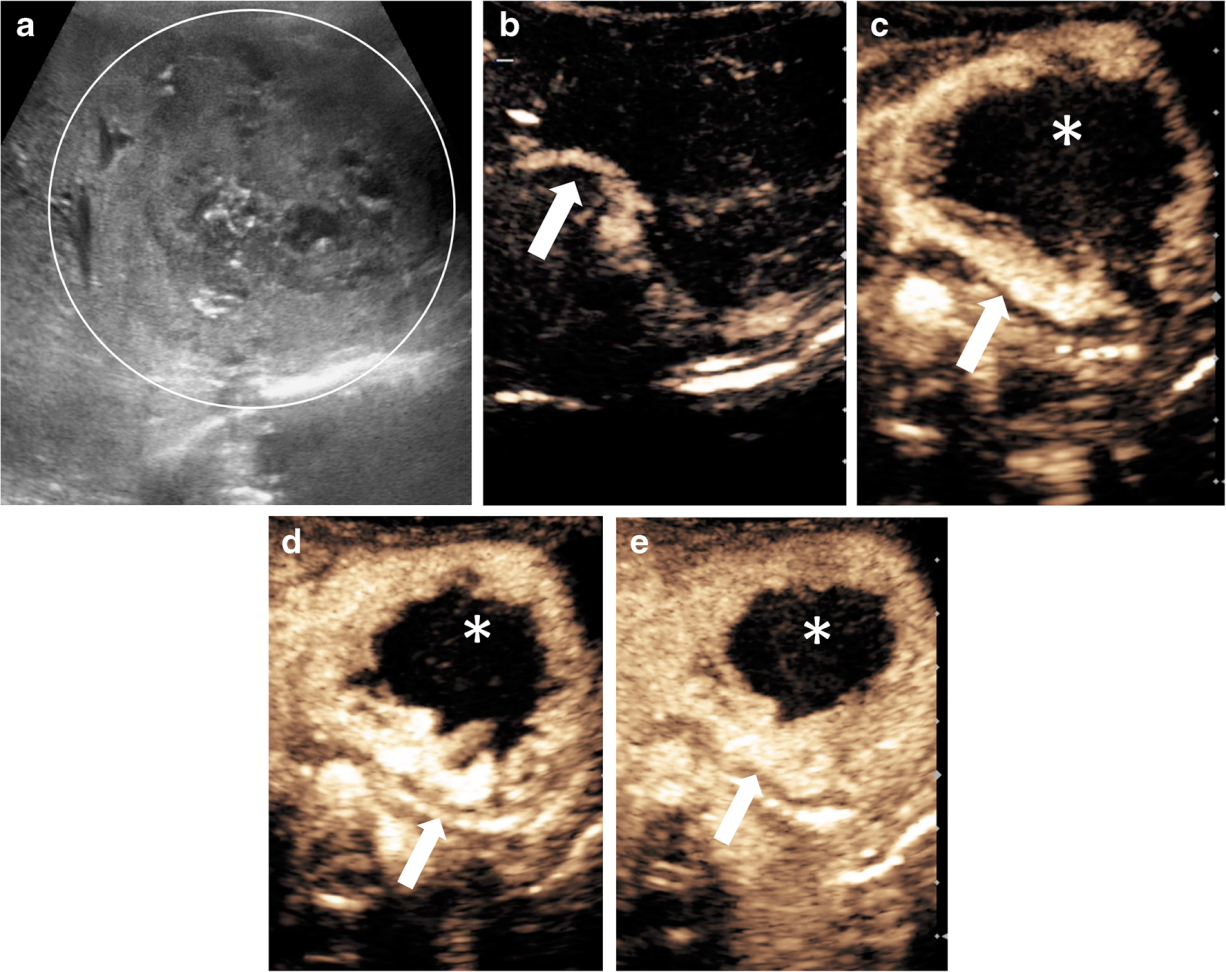
Rapidly involuting congenital hemangioma in a 3-week-old boy with a congenital mass of the left liver lobe. **a** Transverse gray-scale US shows a large heterogeneous lesion (*circle*) within the left liver lobe. The volume of the lesion at initial scan was 41 mL. **b–e** Transverse contrast-enhanced ultrasound (CEUS) of the liver in contrast-only mode in the arterial phase at 11 s (**b**), 14 s (**c**) and 18 s (**d**), and in the portal venous phase 60 s after injection of the contrast agent (**e**). Rapid nodular enhancement progresses from the periphery (*arrows*) to the center, with incomplete enhancement in the central aspect (*asterisks*) of the lesion. In the follow-up US scans (not shown) except for residual calcifications there was near-complete regression of the lesion. Imaging features are typical for rapidly involuting congenital hemangioma. In this child, at 9 months of age the volume of the lesion had decreased at 2.6 mL, and at 5 years of age the lesion was barely visible with a measured residual volume of 0.4 mL

In terms of imaging appearances, many congenital hemangiomas are indistinguishable from infantile hemangiomas (Fig. 2) [60]. They are most commonly solitary. On gray-scale US, congenital hemangiomas typically appear as well-circumscribed heterogeneous lesions. They can be hypo- or hyperechoic, containing multiple anechoic vascular spaces on color Doppler [21, 59]. In one recent study including children with congenital hemangiomas, 60% of the lesions were heterogeneous on gray-scale US and 40% of the lesions were hyperechoic. In this study, a heterogeneous appearance was noticed in larger lesions (>3 cm in diameter), whereas smaller lesions (<1 cm in diameter) appeared predominantly hyperechoic [21]. Other distinctive features of congenital hemangiomas include more calcifications (specific for non-involuting congenital hemangiomas and rapidly involuting congenital hemangiomas), larger flow voids like those of an arteriovenous malformation with associated solid components, and less well-defined margins on CT and MRI [58, 60].

**Fig. 3:**
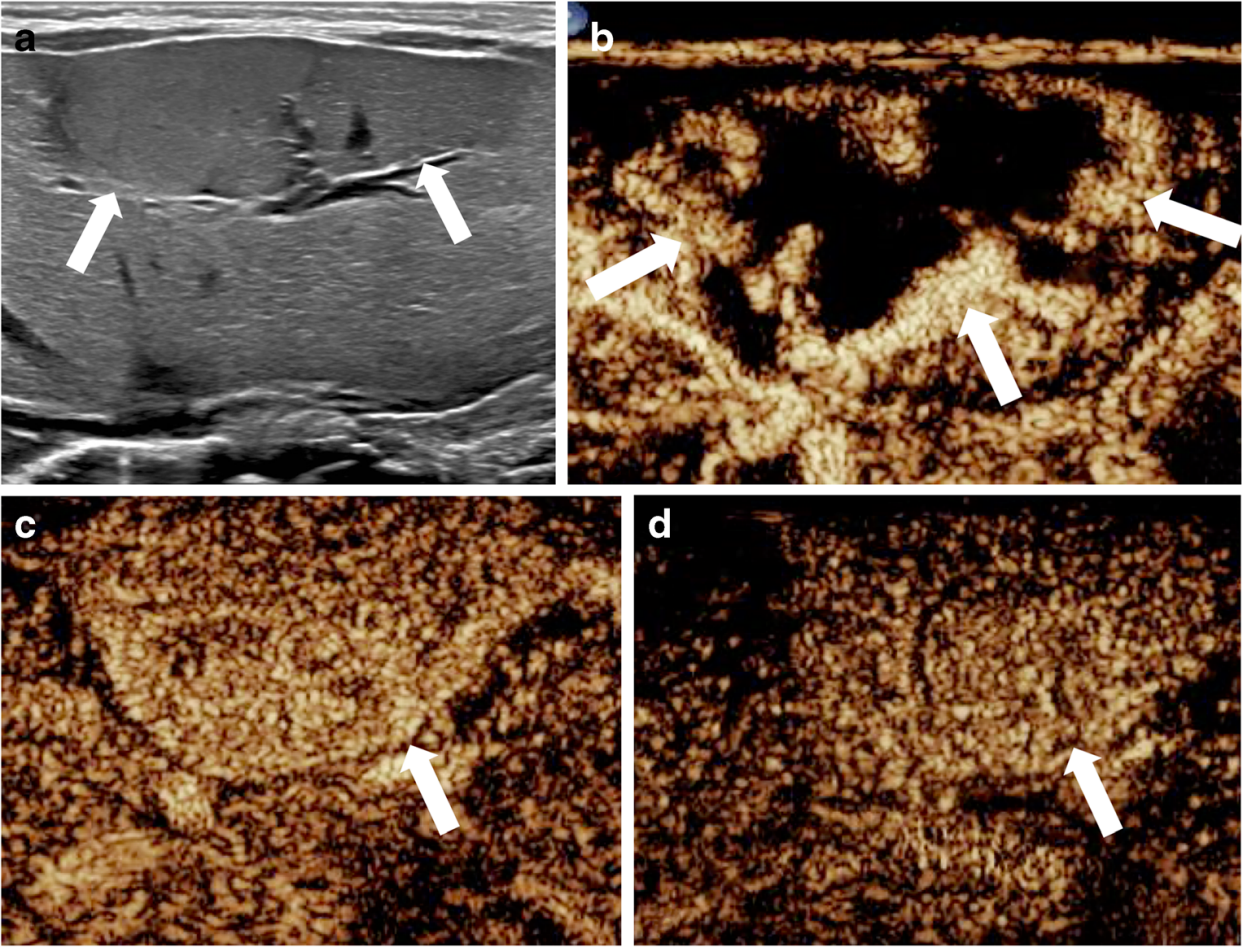
Congenital hemangioma in a 2-week-old boy with elevated liver enzymes. **a** Transverse gray-scale US demonstrates a large focal liver lesion (*arrows*). **b–d** Transverse contrast-enhanced ultrasound (CEUS) images in contrast-only mode during dynamic enhancement phases. In the arterial phase (**b**), the lesion demonstrates peripheral, nodular, discontinuous enhancement pattern (*arrows*). In the portal venous phase (**c**), the lesion (*arrow*) appears homogeneously hyperenhancing. In the late phase (**d**), the lesion (*arrow*) remains hyperenhancing compared to the adjacent liver parenchyma. See Online Supplementary Material 1 for cinematic clip

The CEUS enhancement pattern of the congenital hemangioma is similar to that of infantile hemangioma, demonstrating typically peripheral nodular enhancement during the arterial phase, with centripetal continuous filling during the late portal venous phase (Fig. 3, Online Supplementary Material 1) [57]. In one study including five children with congenital hemangiomas who underwent CEUS, all lesions (100%) showed hyperenhancement in the arterial phase, and most of the lesions (80%) remained hyperenhancing relative to the normal liver parenchyma in the portal venous phase [21]. Larger lesions might have incomplete contrast fill-in [21].

### Focal nodular hyperplasia

Focal nodular hyperplasia (FNH) is a benign tumor that is most frequently found in young to middle-age adults, though it is also encountered in the pediatric population, typically between the ages of 2 years and 5 years. It can also be seen in oncology patients following radiation and chemotherapy [1, 61]. People with FNH are rarely symptomatic, and FNH is often incidentally observed during imaging performed for other reasons.

Focal nodular hyperplasia is thought to represent a proliferative hepatocellular response caused by increased blood flow resulting from a dystrophic artery. Histologically, it is composed of benign-appearing hepatocytes arranged in nodules that are usually partially delineated by fibrous bands originating from the central fibrous scar that contains the dystrophic arterial vessels [62, 63]. Atypical forms of FNH are also recognized, including FNH without a central scar (mostly smaller lesions less than 3 cm in diameter), and FNH with significant steatosis [62, 64]. FNH does not bleed and does not have malignant potential. It can be solitary or multiple and can coexist with other benign or malignant lesions; therefore, accurate identification is crucial.

Because this lesion is composed of normal hepatocytes, on US it appears well-circumscribed and nearly isoechoic with the adjacent normal liver, although it can also be hypo- or, rarely, hyperechoic [65]. The central scar and radiating septa typically appear hyperechoic relative to the remainder of the lesion. Color Doppler US demonstrates radial arterial vascularity of the stellate scar in a spoke-wheel pattern [1].

**Fig. 4:**
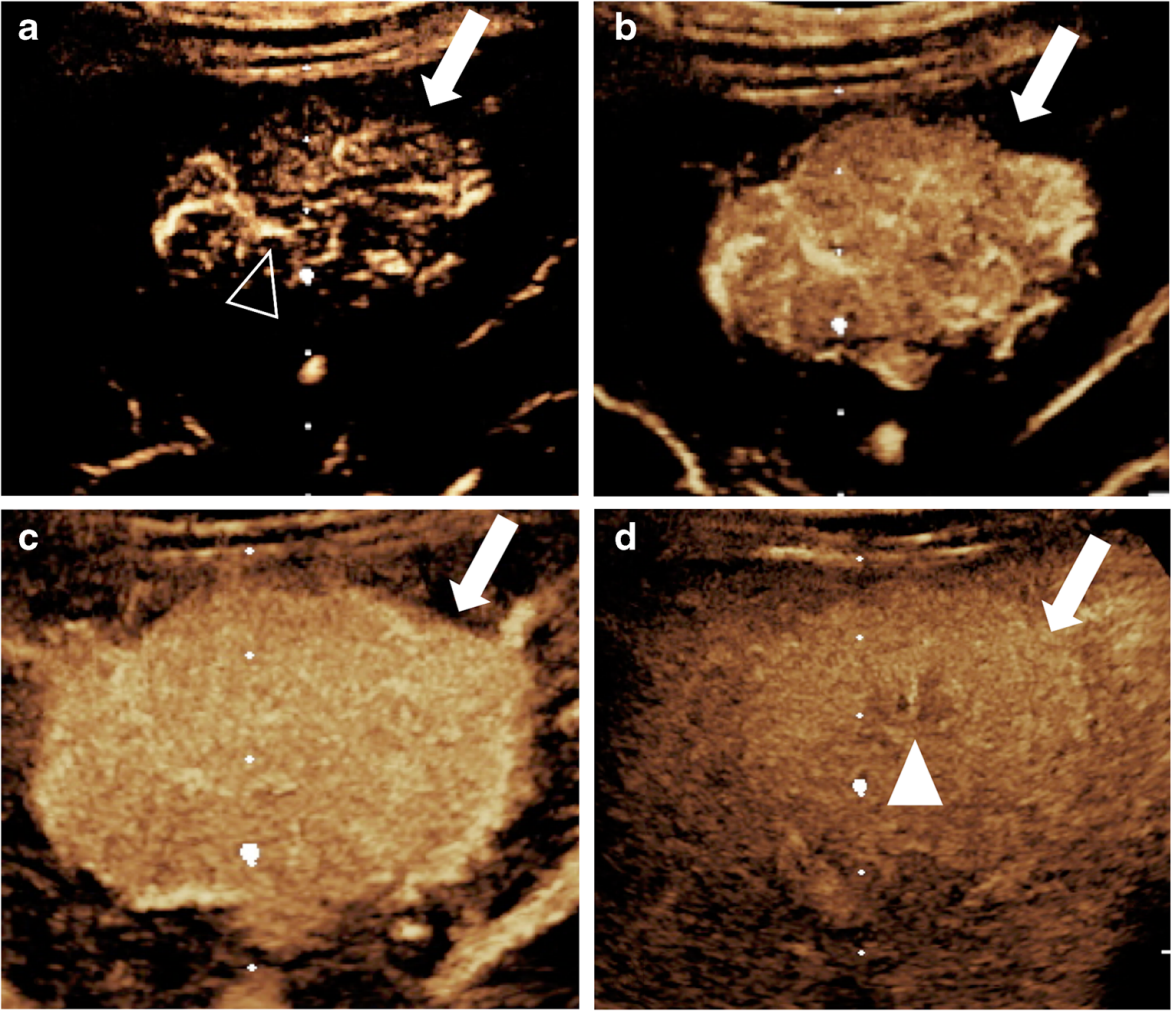
Focal nodular hyperplasia in a 17-year-old boy with abdominal pain. An incidentally detected focal liver lesion was found at gray-scale US (not shown here). **a–d** Transverse contrast-enhanced US images, in contrast-only mode during dynamic enhancement phases. In the early (**a**) and late (**b**) arterial phases, there is rapid spoke-wheel enhancement of the lesion (*arrows*) originating from the center (*arrowhead*). In the portal venous phase (**c**), the lesion (*arrows*) appears homogeneously hyperenhancing and remains hyperenhancing during the late phase (**d**), where a central hypoenhancing scar (*arrowhead*) is evident

In a recent pediatric CEUS study, classic FNH showed the presence of a central feeding artery that is visible in the early arterial phase and demonstrates a centrifugal filling pattern, with stellate (spoke-wheel) hyperenhancement in the arterial to early portal venous phases, and iso- or hyperenhancement in the late portal venous phase (Fig. 4, Online Supplementary Material 2) [7, 66].

There are some contradictory results regarding the correlation between the lesion size and the identification of the central stellate scar on CEUS. Some investigators showed that the classic spoke-wheel pattern depended on the size of the lesion and was more frequently seen in larger FNHs, typically those greater than 3 cm [67, 68]. Other investigators reported that the spoke-wheel pattern was not size-dependent and that centrifugal filling was seen more frequently in small FNHs (less than 3 cm in size) [67–70].

### Hepatic adenomas

Hepatic adenomas are benign, typically solitary lesions that are frequently diagnosed in women ages 35–40 years. Although hepatic adenomas are very rare in children, they are encountered more often in young girls who take oral contraception, in children who are on steroid therapy and in those with underlying metabolic disease such as galactosemia and glycogen storage disease [62, 71]. A hepatic adenoma is composed of benign-appearing hepatocytes, which might contain increased amounts of fat and glycogen [14]. Hepatic adenoma demonstrates a propensity to hemorrhage, particularly if large or exophytic in location; it rarely becomes malignant [72, 73]. For these reasons hepatic adenomas are commonly treated with surgical resection.

On imaging, the appearance of hepatic adenoma depends on the composition of the lesion and of the surrounding liver, as well as the presence of associated complications (e.g.*,* intralesional necrosis and hemorrhage). Uncomplicated hepatic adenomas on US tend to be homogeneous lesions with similar reflectivity to the adjacent normal liver, or they appear relatively hyperechoic if there is high intralesional lipid content. However, on a background of diffuse fatty liver infiltration or glycogen storage disease, adenomas might appear hypoechoic [1]. On color Doppler imaging, hepatic adenomas usually demonstrate multiple vascular channels located peripherally and centrally, with predominately venous flow [74]. Large hepatic adenomas, particularly those greater than 5 cm, can be complicated with rupture and hemorrhage, which is most commonly intralesional; however, in rare instances intraperitoneal hemorrhage and hypovolemic shock occur [1].

**Fig. 5:**
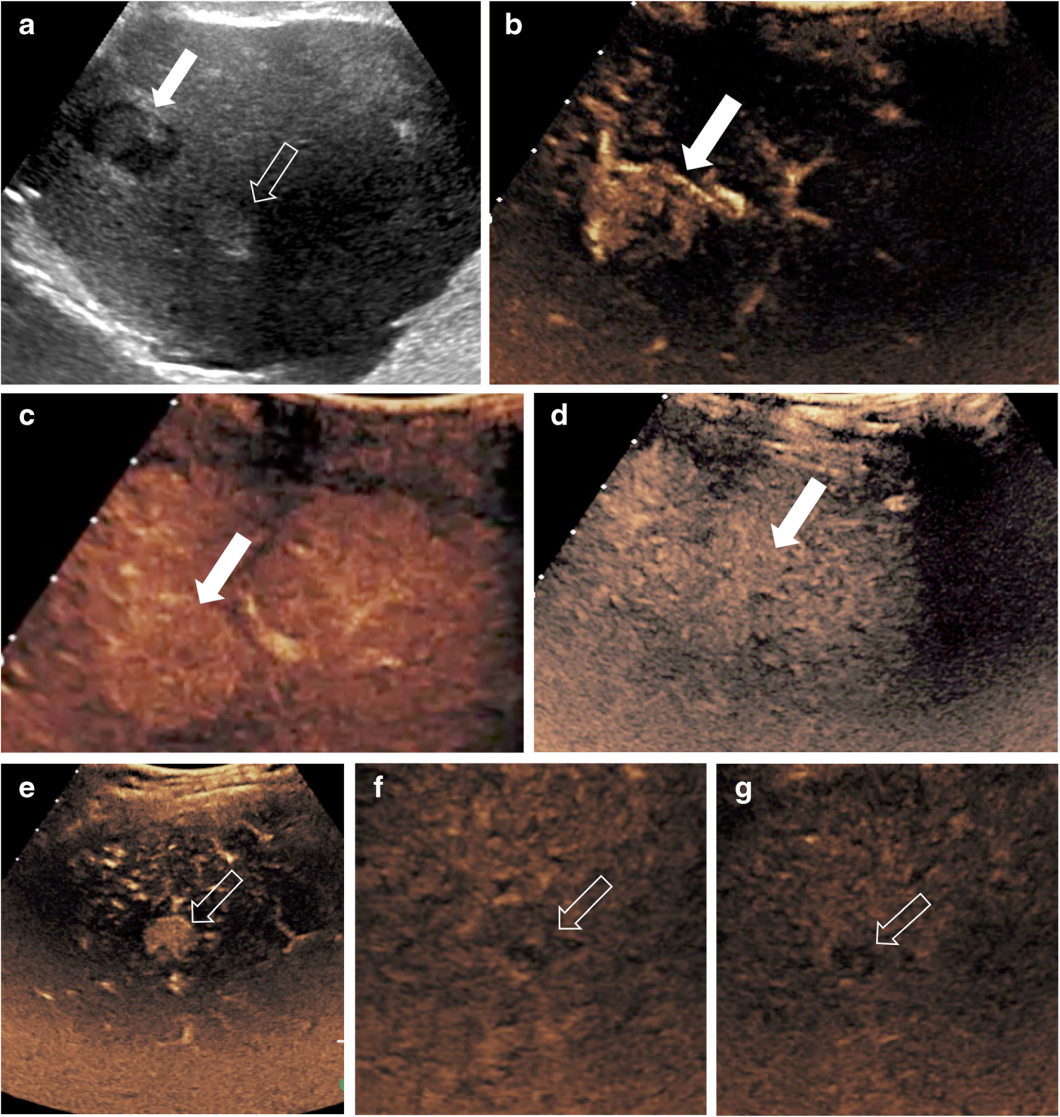
Hepatic adenoma and hepatocellular carcinoma in a 17-year-old girl with glycogen storage disease type 1 who was known to have multiple hepatic adenomas. **a** Transverse gray-scale US demonstrates two focal liver lesions. The larger lesion (*solid arrow*) appears with heterogeneous echotexture but is predominantly hypoechoic, and the smaller lesion (*open arrow*) is hyperechoic. Both lesions were further evaluated with contrast-enhanced ultrasound (CEUS). **b–d** Transverse CEUS images of the first lesion (*arrows*) in contrast-only mode demonstrate hyperenhancement in the arterial phase (**b**), iso- to slightly hyperenhancement in the portal venous phase (**c**) and isoenhancement to background liver in the late phase (**d**). Histological diagnosis: hepatic adenoma. **e–g** Transverse CEUS in contrast-only mode of the second lesion (*arrows*) demonstrates hyperenhancement in the arterial phase (**e**) and hypoenhancement in the portal venous (**f**) and late (**g**) phases. Histological diagnosis: hepatocellular carcinoma

On CEUS, hepatic adenomas typically display subcapsular feeding arteries, resulting in the following perfusion pattern: rapid enhancement in the early arterial phase; homogeneous, complete or near-complete enhancement in the portal phase; and isoenhancement or, rarely, hyperenhancement that usually persists during the delayed phase, although slow progressive hypoenhancement has been reported (Fig. 5) [5, 62, 75–77]. Areas of intralesional hemorrhage appear as non-enhancing regions on CEUS.

### Focal fat or focal fatty sparing

Fatty infiltration is defined as an excessive accumulation of fat within the hepatocytes. This process might not be uniform throughout the liver. Areas with less fat infiltration (spared) or increased fat deposition (focal or diffuse) can mimic liver lesions. Focal fatty changes are often located near the gallbladder fossa, in segment IV (near the falciform ligament) and at the bifurcation of the main portal vein [78].

On gray-scale US, focal fatty infiltration can appear as a distinct lesion or as less defined geographic areas of hyperechogenicity, whereas fatty sparing appears as geographic regions of hypoechogenicity [9]. In both cases, the echogenicity of the lesion is compared to that of the adjacent liver parenchyma. Although typical features on gray-scale US include wedge-shape appearance, location near gallbladder and falciform ligament, and penetration by branches of portal or hepatic veins, occasionally these regions raise concern for focal masses [78, 79].

**Fig. 6:**
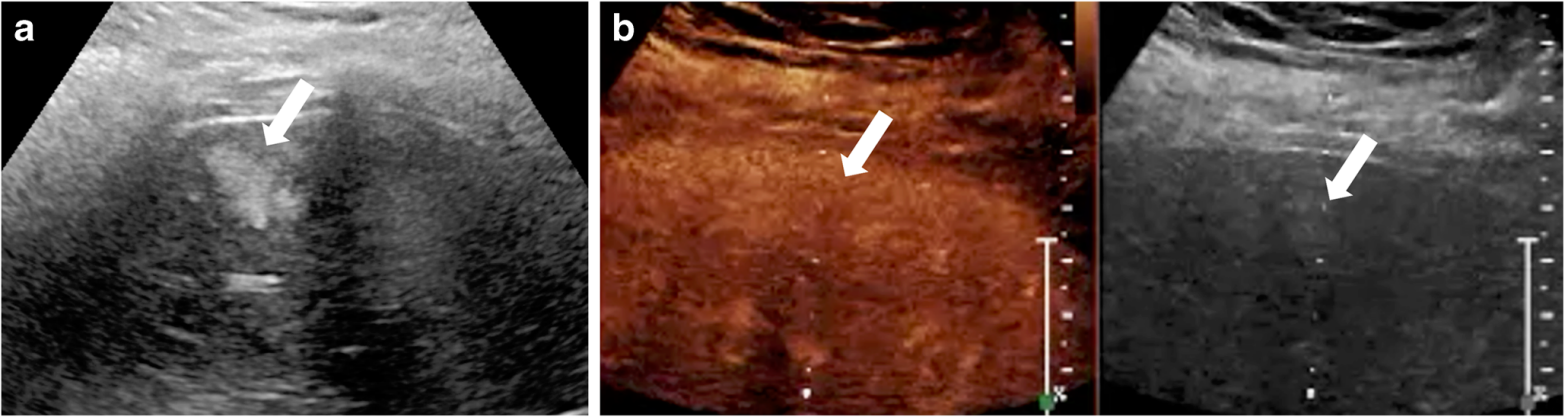
Focal fatty infiltration of the liver in a 16-year-old girl who presented with elevated liver enzymes. **a** Transverse gray-scale US demonstrates a focal hyperechoic lesion (*arrow*) within the subcapsular region of the liver. **b** Contrast-enhanced ultrasound (CEUS) of the liver in transverse plane with dual display of gray-scale (*right*) and contrast-enhanced (*left*) images. The lesion (*arrows*) on gray-scale US shows isoenhancement to the liver background on CEUS in the portal venous phase. It was isoenhanced in all other phases, too (not shown)

On CEUS following UCA administration, areas of focal fat or fatty sparing are expected to enhance in a similar manner as the background liver, with no evidence of abnormal vessels (Fig. 6) [76].

One previous study evaluating the characteristics of focal fatty infiltration of 25 lesions in adults reported a variable frequency pattern in the arterial phase, including isoenhancement (44%), hyperenhancement (12%) and hypoenhancement (44%). In the portal venous phase all lesions (100%) demonstrated isoenhancement, and these areas could not be distinguished from adjacent liver tissue [80]. This variable enhancement in the arterial phase might be explained by altered portal flow dynamics; it was previously shown that areas of fatty liver infiltration adjacent to the liver hilum are supplied by a venous network that originates from the pancreatico-duodenal and pyloroduodenal veins rather than the portal vein [80]. Another explanation might be that extrinsic narrowing of the supplying blood vessels by the adjacent fatty infiltrated hepatic cells affects the degree of UCA enhancement.

### Regenerative nodules

Regenerative nodules, also known as “nodular regenerative hyperplasia,” occur as a hyperplastic response of hepatocytes to an insult. In the rare cases that they occur in children, they are often associated with conditions leading to chronic liver disease (e.g., myelo- and lymphoproliferative disorders, autoimmune disorders, collagen vascular disease) or to previous use of steroids and antineoplastic medication [14].

The appearance of regenerative nodules on US is variable and depends on the size of the nodules. Small nodules might not be apparent, but if there are many of them the liver appears with diffusely heterogeneous echotexture or distortion of normal architecture. If nodules are visible, they are generally well-circumscribed, homogeneous, and hypoechoic or hyperechoic compared with the normal liver [14, 81].

On CEUS these nodules show similar enhancement with the background liver parenchyma throughout all the enhancement phases. They are distinct from their malignant counterparts in that they do not show washout during the portal venous or late phases [82].

## Interpretation of contrast-enhanced ultrasound findings in hepatic malignancy

Among the primary and secondary malignant hepatic tumors known in children are hepatoblastoma, hepatocellular carcinoma, undifferentiated (embryonal) sarcoma, angiosarcoma, embryonal rhabdomyosarcoma, lymphoma and metastasis. Their appearance is variable on gray-scale US and can overlap with that of benign lesions. A recent meta-analysis of 57 studies showed that CEUS has excellent diagnostic accuracy in differentiating malignant from benign focal liver lesions, with a pooled sensitivity and specificity of 0.92 (95% confidence interval [CI]: 0.91–0.93) and 0.87 (95% CI: 0.86–0.88), respectively [83]. The average age of the patients in the studies included in this meta-analysis ranged from 13 years to 70 years; however, only one study was exclusively in pediatric patients [9]. The hallmark of both primary and metastatic malignant liver lesions is the washout of the contrast agent in any given phase, occurring mostly during the portal venous or delayed phases [42].

The arterial-phase enhancement pattern of malignant lesions can be variable. For example, hypervascular metastasis and hepatocellular carcinoma exhibit hyperenhancement during the arterial phase, while some metastases appear as hypoenhanced or demonstrate a rim-like enhancement in the arterial phase. Larger malignant lesions might show a disorganized internal vasculature, which becomes more visible on CEUS studies than it does on non-contrast ultrasound, CT or MRI.

It is important to consider that the enhancement pattern of hepatic inflammatory and infectious lesions on CEUS can be like those of primary liver malignancy and metastatic disease. Because of the hyperemia associated with infection, abscesses have a peripheral rim of hyperenhancement during the arterial phase of CEUS. If the central component is replaced by purulent material, then the center does not enhance. However, depending on the degree of liquefaction the central aspect might also be hypoenhancing or have a well-defined non-enhancing region surrounded by enhancing septations. During the venous phase, inflammatory and infectious lesions show hypoenhancement. These patterns can be indistinguishable from malignant lesions, and biopsy might be required if the history, clinical signs and symptoms are not clear [84, 85].

### Hepatoblastoma

Hepatoblastoma is the most common primary malignant pediatric liver tumor, accounting for more than 90% of liver cancers in children younger than 5 years. It usually occurs sporadically, but it is sometimes associated with prematurity, Beckwith–Wiedemann syndrome, hemihypertrophy and familial adenomatous polyposis coli [2, 86]. It presents with nonspecific abdominal symptoms, but the most common clinical presentation is a palpable abdominal mass; only occasionally acute abdominal pain from intratumoral hemorrhage or rupture is encountered.

Hepatoblastoma is a well-circumscribed, encapsulated, large solid mass with a lobulated contour. Epithelial hepatoblastomas typically demonstrate a relatively more homogeneous appearance, while mixed epithelial and mesenchymal tumors appear markedly heterogeneous. Teratoid components appear cystic. Areas of hemorrhage or necrosis within the tumor are common [3]. Coarse calcifications are present in 20–50% cases.

**Fig. 7:**
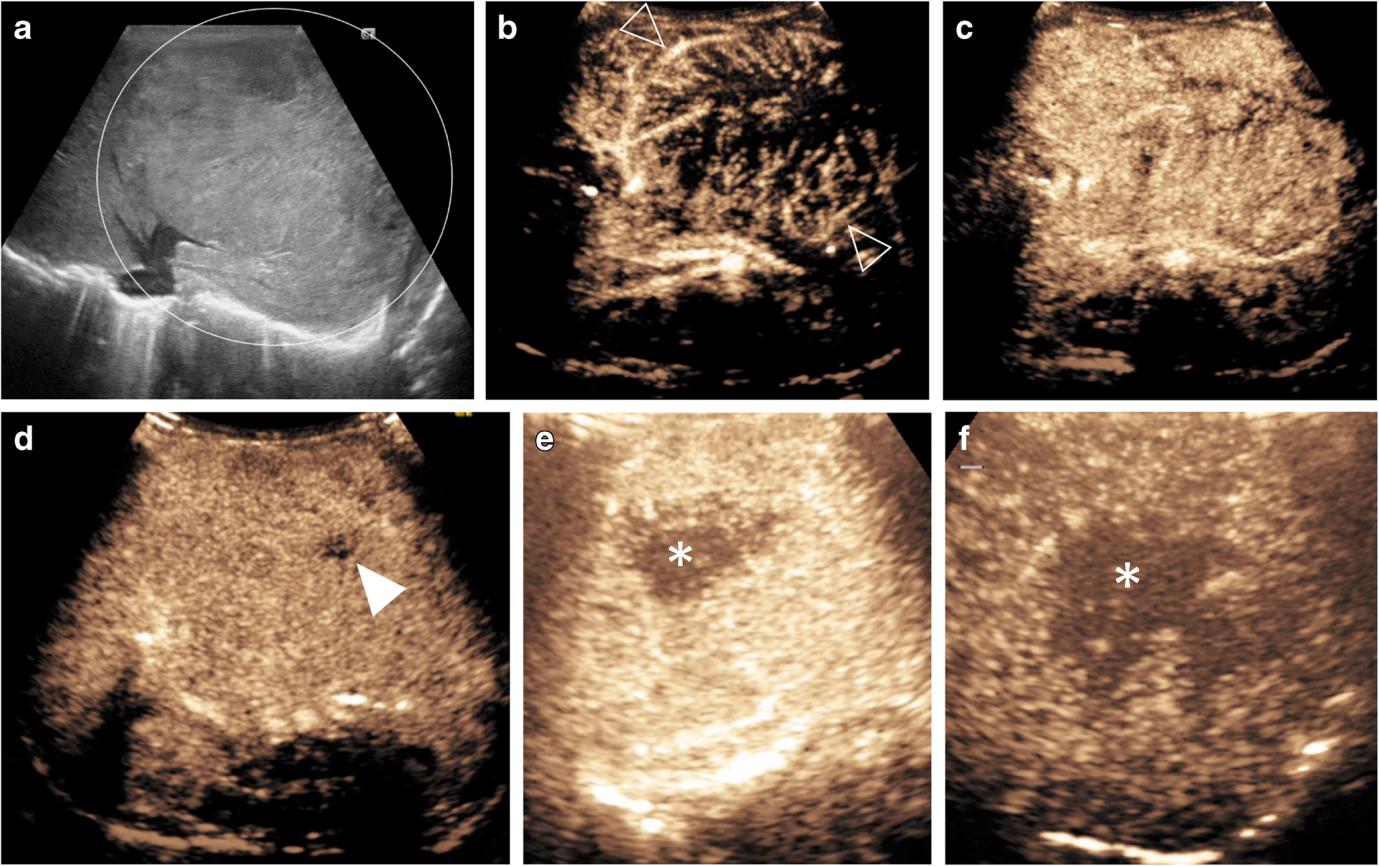
Hepatoblastoma in an 11-day-old boy with a large congenital mass of the left liver lobe. **a** Gray-scale US shows a large mass with heterogeneous echotexture (*circle*) within the left liver lobe. **b–f** Contrast-enhanced ultrasound (CEUS) of the liver. Transverse CEUS in contrast-only mode in arterial phase at 10 s (**b**) and 14 s (**c**), in portal venous phase at 53 s (**d**) and in late-phase of enhancement >120 s after contrast injection (**e**, **f**). Rapid heterogeneous enhancement shows multiple irregular vessels (*open arrowheads*) centrally within the lesion in a disorganized pattern. One small region in the periphery of the lesion (*solid arrowhead*) shows persistent reduced enhancement, possibly corresponding to necrosis. Transverse CEUS during the late phase (**e** and later **f**) shows progressive washout (*asterisks*) centrally

On CEUS hepatoblastoma can show early peripheral enhancement during the arterial phase and marked contrast washout during the late portal venous phase (Fig. 7) [4]. It can invade the portal or hepatic veins, and when this happens CEUS can accurately identify a tumor thrombus as an enhancing filling defect within the invaded vessel.

### Hepatocellular carcinoma

Hepatocellular carcinoma (HCC) is the second most common primary malignant liver tumor in children, accounting for 20–30% of primary hepatic malignancies. It is usually found in older children and adolescents (10–14 years) but has also been found in children younger than 5 years. Several diseases in children can lead to cirrhosis, which increases the risk of developing HCC (e.g., glycogen storage diseases, progressive familial intrahepatic cholestasis, biliary atresia, Alagille syndrome and Fontan hepatopathy) [87]. However, most cases of HCC occur in children without underlying liver disease [87, 88]. Additionally, there are two variants of HCC in the pediatric age group: the first is hepatocellular malignant neoplasm not otherwise specified (previously referred to as transitional liver cell tumor), which is composed of both hepatoblastoma and HCC components; the second is fibrolamellar HCC, which typically presents with normal alpha-fetoprotein levels and accounts for almost 30% of HCCs in people younger than 20 years [89–91].

The enhancement patterns of HCC on CEUS have been detailed in adults using the CEUS Liver Imaging Reporting and Data System (LI-RADS) [45]. This system characterizes a lesion as HCC using various visual CEUS imaging features: the lesion’s size; the type and degree of arterial phase enhancement; and the presence, onset and degree of washout.

On CEUS, HCC typically demonstrates arterial-phase hyperenhancement, which can involve diffusely the whole lesion or a portion of the lesion (in what is known as nodule-in-nodule or mosaic appearance). Another typical feature is washout, which is late in onset (beginning 60 s after contrast injection) and mild in degree (the lesion becomes less enhanced than the surrounding liver but still demonstrates some degree of persistent contrast enhancement within 2 min after contrast injection) [45]. The CEUS imaging appearance is likely similar in children. Although fibrolamellar HCC is distinct from HCC, a case report of a CEUS in a child with fibrolamellar HCC described arterial hyperenhancement followed by washout of the tumor beginning approximately 40 s after contrast injection; hyperenhancement was also noted within the tumor thrombus in the portal vein (Online Supplementary Material 3) [92]. If there is early onset or marked washout, this suggests a malignant tumor other than HCC (Fig. 5).

### Metastatic disease

A common feature of all metastatic liver lesions on CEUS is contrast washout, which is typically rapid (beginning within 60 s after contrast injection) and marked (the lesion is completely devoid of contrast within 2 min after contrast injection), resulting in a “punched-out” appearance (Online Supplementary Material 4) [45]. In the arterial phase, however, metastasis can appear as hyperenhancing or hypoenhancing lesions with variable patterns, including homogeneous, rim-like or heterogeneous patterns [49, 93].

Differentiating between benign cystic lesions and cystic hepatic metastasis can be challenging on gray-scale imaging. On CEUS, though, the distinction is clearer: cystic hepatic metastasis might show peripheral rim-like hyperenhancement in the arterial phase, while benign cysts usually do not show any enhancement [94]. However, unlike benign cystic lesions, all cystic metastatic lesions show contrast washout of the enhancing areas during the portal venous or the late venous phases [94, 95]. When CEUS was used in addition to gray-scale US to distinguish between benign (hemorrhagic cyst, hemangiomas) and malignant cyst-like focal liver lesions, the sensitivity, specificity, and positive and negative predictive values all increased significantly. Similarly, when CEUS was used to assess focal liver lesions, better interobserver agreement was reported [95].

### Other malignant hepatic lesions

Trenker et al. [96] documented CEUS appearances of hepatic lymphoma in a cohort of 38 adults. In their study, lymphoma was hypoechoic on gray-scale imaging in 97.4% of cases and demonstrated variable enhancement in the arterial phase with 23.7%, 44.7% and 31.6% of lesions appearing hyper-, iso- and hypoenhancing, respectively. When compared to the background liver, 94.7% were hypoenhancing in the portal venous phase and all were hypoenhancing in the late parenchymal phase. The lack of a consistent enhancement pattern makes it impossible to definitively distinguish hepatic lymphoma from other solid malignant lesions. However, the fact that lymphomas do not tend to distort the native vessels can be used as a sign to differentiate them from other malignant tumors.

## Comparative studies

The safety and diagnostic accuracy of CEUS for characterizing focal liver lesions in adults have been well established. A prospective multicenter study of 1,349 mostly adult patients showed that CEUS has a 95.8% sensitivity in identifying malignant lesions [97]. The diagnostic performance of CEUS is described in Table 2 [66, 67, 75, 83, 97–104]. However, at this point the studies for hepatic CEUS application in children are relatively limited.

**Table 2:** Contrast-enhanced ultrasound (CEUS) diagnostic performance

Reference^a^	Age range (years)	Subjects (*n*)	CEUS application	CEUS performance
Meta-analysis				
Wu 2018 [83]^b^	13–70	57^c^	FLL	Pooled: Sn 0.92; Sp 0.87; OR 104.20
Prospective studies				
Strobel 2011 [98]	12–91	1,349	Small FLLs ≤20 mm	Acc 83.8%; Sn 93.5%; Sp 66.7%; PPV 92.3%; NPV 95.1%
Strobel 2009 [99]	12–91	1,349	Vascular characteristics of FLLs	Acc 83.1% benign lesions Acc 95.8% malignant lesions Acc 91.4% liver metastases Acc 84.9% HCC
Strobel 2008 [97]	12–91	1,349	Differentiation of FLLs	Acc 90.3%; Sn 95.8%; Sp 83.1%; PPV 95.4%; NPV 95.7%
Ungermann 2007 [67]	17–81	28	Enhancement pattern of FNH	Stellate enhancement in 95% lesions >3 cm and 30% <3 cm; central scar in 85% lesions >3 cm and 20% <3 cm
Wu 2006 [100]	16–78	79	Role to localize FLLs for biopsy and diagnostic accuracy of biopsy	Acc biopsy significantly higher with CEUS than US, 95.3% vs. 87.4%, respectively; Acc biopsy malignant lesions significantly higher with CEUS than US, 97.1% vs. 78.8%, respectively
Retrospective studies				
Kong 2015 [75]	15–54	38	Enhancement pattern FNH and hepatic adenoma	39.5% of lesions were correctly assessed with color Doppler US alone; 65.8% of lesions were correctly assessed with color Doppler US and CEUS
Pei 2012 [101]	15–75	130	Quantitative perfusion analysis of HCCs	Washout time faster for poorly differentiated HCCs than for moderately and well-differentiated HCCs
Rennert 2011 [102]	0.3–85.0	100	Image fusion and surgical planning for FLLs	Image fusion allowed identification of additional lesions in 12 patients, changing management
Kim 2008 [66]^d^	14–81	62	Differentiate FNH and hepatic adenoma	Sn 95% and 86%; Sp 74% and 79%; PPV 89% and 90%; NPV 88% and 71%, for two readers, respectively
Wang 2008 [103]	17–86	52	Undetermined FLL in fatty liver	Acc 91%; Sn 91.7%; Sp 90.9%
Soye 2007 [104]	17–83	68	Characterize FLL	Sn 95%; Sp 97.9%

*Acc* accuracy, *FLL* focal liver lesion, *FNH* focal nodular hyperplasia, *HCC* hepatocellular carcinoma, *NPV* negative predictive value, *OR* odds ratio, *PPV* positive predictive value, *Sn* sensitivity, *Sp* specificity

^a^Original research, contrast-enhanced ultrasound (CEUS) studies including a mixed population of children and adults for focal liver lesions. All studies used SonoVue except where indicated

^b^Included studies used SonoVue, Sonazoid (GE Healthcare, Milwaukee, WI), Optison and Definity

^c^Number of CEUS studies. In all other studies the number of subjects is presented

^d^Contrast agent not specified

In children, one study demonstrated that CEUS had a 98% specificity and 100% negative predictive value for distinguishing benign from malignant liver lesions, using CT, MRI or histology as reference standards [9]. In that cohort of 44 children, only one lesion was falsely identified as being malignant on CEUS as well as on CT and MRI; however, histology at the time of resection indicated that it was a hepatic adenoma [9]. In another pediatric CEUS study, the investigators reported that CEUS had a sensitivity of 82%, specificity of 100%, negative predictive value (NPV) of 88% and positive predictive value (PPV) of 100% for correctly differentiating between benign and malignant lesions, using CT, MRI or histology as reference standards [15]. In a cohort of 30 children with definitive or probable hepatic adenoma and FNH, CEUS features were concordant with histology and MRI in 77.8% and 67.9% of cases, respectively; those investigators showed that by combining findings from MRI and CEUS, the proportion of lesions that could be classified as FNH or hepatic adenoma increased to 96.4% [7]. Most important, performing CEUS as the initial imaging examination can help avoid further imaging with CT or MR or even prevent the need for a biopsy. Yusuf et al. [8] conducted CEUS of the liver in 147 pediatric patients and were able to avoid any further diagnostic or interventional procedure in 73 (49.7%) of them.

## Limitations

One disadvantage of CEUS when compared to CT and MR is that CEUS is more operator-dependent. Therefore, diagnostic accuracy is limited by the operator’s ability to interrogate the abnormality. Another challenge when using CEUS is the inability to assess multiple lesions in the liver when they are not visible within a single field of view. However, this obstacle can be overcome either by evaluating additional lesions with subsequent injections of UCA or with transducer sweeps through the liver in the transverse and sagittal planes after the dynamic phase. These sweeps can identify lesions as they demonstrate washout, which is the key feature of malignancy.

## Conclusion

Recent FDA approval of Lumason for intravenous liver applications in children has facilitated the widespread use of CEUS in the evaluation of focal liver lesions. CEUS is the ideal adjunct to gray-scale US when focal liver lesions are indeterminate. CEUS, with its ability to accurately distinguish between benign and malignant lesions, improves patient management by enabling a definite diagnosis in most cases. In fact, CEUS is so effective that it can often replace other cross-sectional imaging or interventional procedures. Although much more research is to be done on pediatric applications, CEUS promises ease and accuracy in the clinical setting.

## Supplementary Information

Online Supplementary Material 1 A 2-week-old boy with elevated liver enzymes. Same patient as Fig. 3. Transverse contrast-enhanced ultrasound (CEUS) cinematic clip with dual display of gray-scale (*right*) and contrast (*left*) images. In the arterial phase, the lesion demonstrates a peripheral, nodular, discontinuous enhancement pattern. In the portal venous phase, the lesion appears homogeneously hyperenhancing, whereas in the late phase, the lesion remains hyperenhancing compared to the adjacent liver parenchyma (MP4 7,890 kb)

Online Supplementary Material 2 A 17-year-old girl with abdominal pain. Focal liver lesion was identified on US that was performed for appendicitis. Sagittal contrastenhanced ultrasound (CEUS) cinematic clip of the liver with dual display of gray-scale (*left*) and contrast (*right*) images. During the early arterial phases, rapid enhancement of the lesion originates from the center and progresses centrifugally in in a spoke-wheel pattern. In the portal venous phase, the lesion is homogeneously hyperenhancing and remains hyperenhancing during the late phase, where a central hypoenhancing scar is evident. This enhancement pattern is in keeping with focal nodular hyperplasia

Online Supplementary Material 3 A 14-year-old boy with fibrolamellar hepatocellular carcinoma (HCC). Sagittal contrast-enhanced ultrasound (CEUS) cinematic clip of the right liver lobe with dual display of gray-scale (*left*) and contrast-enhanced (*right*) images. There is hyperenhancement of the lesion in the early arterial phase followed by isoenhancement and slow washout. These contrast-enhanced US features are typical of HCC (MP4 6,657 kb)

Online Supplementary Material 4 A 7-year-old girl with Wilms tumor and metastatic liver disease. Sagittal contrast-enhanced ultrasound (CEUS) cinematic clip of the liver with dual display of gray-scale (*left*) and contrast-enhanced (*right*) images. There is early washout of the lesion beginning at about 10 s. Early washout is typical of liver metastases on CEUS (MP4 7,345 kb)
